# Distribution of Brevetoxin (PbTx-3) in Mouse Plasma: Association with High-Density Lipoproteins

**DOI:** 10.1289/ehp.8010

**Published:** 2005-06-23

**Authors:** Ricky T. Woofter, Page C. Spiess, John S. Ramsdell

**Affiliations:** Marine Biotoxins Program, Center for Coastal Environmental Health and Biomolecular Research, National Oceanic and Atmospheric Administration–National Ocean Service, Charleston, South Carolina, USA

**Keywords:** blood, brevetoxin, ciguatoxin, HDL, lipoprotein, plasma

## Abstract

We investigated the brevetoxin congener PbTx-3 to determine its distribution among carrier proteins, including albumin and blood lipoproteins. Using a radiolabeled brevetoxin tracer (PbTx-3), we found that 39% of the radiolabel remained associated with components in mouse plasma after > 15 kDa cutoff dialysis. Of this portion, only 6.8% was bound to serum albumin. We also examined the binding of brevetoxin to various lipoprotein fractions. Plasma, either spiked with PbTx-3 or from mice treated for 30 min with PbTx-3, was fractionated into different-sized lipoproteins by iodixanol gradient ultracentrifugation. Each fraction was then characterized and quantified by agarose gel electrophoresis and brevetoxin radioimmunoassay, respectively. In both the *in vitro* and *in vivo* experiments, the majority of brevetoxin immunoreactivity was restricted to only those gradient fractions that contained high-density lipoproteins (HDLs). Independent confirmation of brevetoxin binding to HDLs was provided by high molecular weight (100 kDa cutoff) dialysis of [^3^H]PbTx-3 from lipoprotein fractions as well as a scintillation proximity assay using [^3^H]PbTx-3 and purified human HDLs. This information on the association of brevetoxins with HDLs provides a new foundation for understanding the process by which the toxin is delivered to and removed from tissues and may permit more effective therapeutic measures to treat intoxication from brevetoxins and the related ciguatoxins.

Red tides have been documented on the Gulf Coast of Florida as early as 1530 (Taylor 1917). They occur almost annually and often persist for many months (Woodcock 1948). Red tides are caused by the dinoflagellate *Karenia brevis* (formerly *Gymnodinium breve* and *Ptychodiscus brevis*), which produces a series of polycyclic ethers called brevetoxins (PbTx) (Davis 1948; Lin et al. 1981; Martin and Chatterjee 1969; Poli et al. 1986 ). These events are responsible for fish, waterfowl, and marine mammal mortalities (Landsberg, 2002; Landsberg and Steidinger 1998) as well as for human intoxication. Aerosol forms of the toxin are produced by wind and wave action and move inshore, causing transient respiratory irritation in people that inhale the toxin (Pierce 1990). Humans can also experience the more severe symptoms of neurotoxic shellfish poisoning (NSP) as a result of consuming shellfish that have accumulated brevetoxin (McFarren et al. 1965).

The action of brevetoxins on its pharmacologic target, the voltage-sensitive sodium channel, leads to activation at normal resting potential and inhibition of inactivation (Huang et al. 1984; Sheridan and Adler, 1989; Westerfield et al. 1977). However, before reaching its target tissue, brevetoxin must be absorbed into the general blood supply. Administration of [^3^H]PbTx-3 orally to rats leads to accumulation of toxin in the liver, stomach, and intestines (Cattet and Geraci 1992). Fat-soluble substances, such as brevetoxins, coalesce into fat droplets in the stomach and are emulsified into mixed micelles by the action of bile acids secreted into the intestine. The mixed micelles are likely absorbed via phagocytosis by absorptive cells of the intestinal villi. Toxin then enters lymph vessels that drain to the subclavian vein to enter the liver via the portal circulation. In the liver, fat-soluble toxicants are in part detoxified by a two-step process that conjugates a polar entity that is released back into the intestine, where it may be reabsorbed and eventually eliminated in urine.

Several toxicokinetic studies treating rats with [^3^H]-PbTx3 have shown that blood retains detectable levels of the radioligand (Benson et al. 1999; Cattet and Geraci 1993; Poli et al. 1990b). More recently, studies treating mice, rats, or fish and measuring toxin using receptor assay and radioimmunoassay have quantified blood brevetoxin and determined that substantial levels (20–30 nM) are retained in all three species (Fairey et al. 2001; Radwan et al. 2005; Woofter et al. 2003, 2005). These values are approximately one order of magnitude higher than the concentration of toxin necessary to activate voltage-gated sodium channels in nerve, heart, or muscle (Bottein Dechraoui and Ramsdell 2003). This indicates that a significant fraction of brevetoxin is bound to elements in blood, reducing its availability to be biologically active at the sodium channel. Brevetoxin, like other lipophilic agents, is likely to partition to cellular elements in blood, and brevetoxin immunoreactivity has been reported in tissue lymphocytes and macrophages of manatees poisoned by brevetoxins (Bossart et al. 1998). Additionally, brevetoxins may associate with specialized transport proteins in the plasma.

Blood is technically a tissue containing a fluid matrix known as the plasma. Plasma is a protein-rich solution containing a diverse number of proteins that serve a variety of functions, including transport, immune response, and tissue-to-tissue signaling (Anderson and Anderson 2002). A primary function of plasma is the distribution of insoluble substances through the use of carrier proteins. Albumin is the most abundant plasma protein and serves as a low-affinity, high-capacity binding protein for steroid hormones and delivers these insoluble substances to target cells in the peripheral circulation (Kragh-Hansen 1981). Plasma also includes more highly specialized binding proteins. Perhaps best known are the high-affinity binding proteins for sex steroids, thyroid hormones, and corticosteroids. In addition, the plasma contains a unique lipoprotein transport system for the precursor of these steroid hormones, cholesterol. Lipoproteins, protein-coated fatty acid/cholesterol complexes, distribute cholesterol to tissues and remove cholesterol from the plasma.

Plasma carrier proteins are believed to have evolved, at least in part, to serve a functional role to bind and transport nonendogenous hydrophobic substances (Baker 2002). We examined the binding of brevetoxin to plasma carrier proteins in mice, looking especially at lipoproteins, and evaluated the role of plasma carrier proteins in the distribution of brevetoxins to target tissues and the elimination of brevetoxins from the organism.

## Materials and Methods

### Brevetoxin mouse plasma spike.

Mouse plasma in EDTA was obtained from Harlan Bioproducts (Indianapolis, IN). We spiked a 9.991-mL sample of mouse plasma with 9 μL of 100 μg/mL PbTx-3 to give a concentration of approximately 100 ng/mL PbTx-3 in the mouse plasma. This solution was then covered, vortexed, and stored at 6°C for 2 hr. We added 2 mL of 60% iodixanol {5,5′-[(2-hydroxy-1-3 propanediyl)-bis(acetylamino)] bis [*N,N*′-bis(2,3-dihydroxypropyl-2,4,6-tri-iodo-1,3-benzenecarboxamide)]} to the 10 mL spiked mouse plasma and allowed it to sit for 2 hr at approximately 4°C. All iodixanol solutions were kept wrapped in aluminum foil and stored at 4°C. In two ultracentrifuge tubes, 9% iodixanol solution (5 mL) was underlayed by 5 mL of the spiked mouse plasma in iodixanol. We carefully pipetted 5 mL of HEPES buffered saline on top.

### Brevetoxin mouse exposure.

We obtained 20 female ICR mice, 18–20 g, from Harlan Sprague Dawley (Indianapolis, IN). The mice were kept for 24 hr with food and water given *ad libitum*. We injected 10 of the mice intraperitoneally (ip) with a maximally tolerable dose (310 μg/kg) of PbTx-3 (Calbiochem, La Jolla, CA) in 3.1% methanol in phosphate-buffered saline (PBS). The other 10 mice were injected ip with 3.1% methanol in PBS. The brevetoxin congener (PbTx-3 was chosen for these experiments because it is less reactive to metabolism in rodents than its precursor (PbTx-2) (Radwan et al. 2005). We based the 30-min time point for blood collection on previous toxicokinetic experiments to assure measurable levels of toxin in fractionated plasma by radioimmunoassay (RIA) (Woofter et al. 2003). After a 30-min exposure, the mice were euthanized with carbon dioxide and exsanguinated via cardiac puncture to the left ventricle with a heparinized 1-cc syringe. The blood was collected in two BD Vacutainer CPT tubes (BD Vacutainer Systems, Franklin Lakes, NJ). The tubes were centrifuged for 20 min at 2,100 relative centrifugal force. We then extracted the plasma by pipette and transferred it to culture tubes. Mice were treated in accordance with the *Guidelines for the Care and Use of Laboratory Animals* (Institute of Laboratory Animal Resources 1996), and all possible efforts were made to reduce animal suffering and to minimize the number of animals used.

### Gradient ultracentrifugation.

Two ultracentrifuge tubes containing 5 mL of 9% iodixanol solution were underlayed with 4 mL extracted mouse plasma in 1 mL iodixanol. Five milliliters of HEPES buffered saline was carefully pipetted on top. Samples were centrifuged at 16°C in a Sorvall swinging bucket rotor (Kendro Laboratory Products, Asheville, NC) for 51 hr at 160,000 × *g* (acceleration and deceleration 8). The tubes were extracted without shaking after ultracentrifugation. We punctured the ultracentrifuge tubes 3 cm from the bottom of the tube and collected 0.5-mL fractions. Fractions from the spiked mouse plasma tubes were collected in Eppendorf tubes, and fractions from the injected mice were collected in glass centrifuge tubes.

### Agarose gel electrophoresis lipoprotein analysis.

We used a HYDRAGEL LIPO + Lp(a) K20 gel electrophoresis kit (SEBIA, Norcross, GA) for fraction characterization. Stock buffer (75 mL) was diluted in distilled water to a volume of 1 L. We made the Sudan black staining solution by adding 80 mL pure ethanol, 1 mL Sudan black stock solution, and 70 mL distilled water. This was allowed to stir gently for 30 min before use. A wash solution was prepared by diluting 16 mL of the wash stock solution in distilled water to a volume of 1 L. We used a destaining solution of 45% ethanol in water.

We filled the HYDRAGEL K20 applicator wells with 10 μL of lipoprotein fraction; each applicator was loaded within 2 min. The applicator was lowered onto the applicator carrier holding the gel and was allowed to rest on the gel for 7.5 min before being removed and discarded. We placed the gel into a SEBIA K20 electrophoresis chamber and allowed it to run for 90 min at 50 V. The gels were removed from the chamber and placed in an 80°C oven for 6–10 min or until dry. After being allowed to cool to room temperature, we placed each gel in a gel holder and immersed it in 50 mL Sudan black staining solution for 15 min. Each gel was then placed in 50 mL destaining solution for 5 min and then immersed in 50 mL wash solution for 1 min. The gels were dried at 80°C for 6–10 min and visually characterized. We stored gels for possible later use at approximately 4°C.

### Brevetoxin radioimmunoassay.

We performed radioimmunoassays for detecting brevetoxin using a sheep antisera prepared against a PbTx-2-fetuin conjugate (Garthwaite et al. 2001; Woofter et al. 2001). RIAs were run in 12 × 75 borosilicate glass tubes in PBS containing 137 mM NaCl, 8 mM Na_2_HPO_4_, 1.5 mM KH_2_PO_4_, and 2.7 mM KCl (all from Sigma Chemical Company, St. Louis, MO), and 0.01% Emulphor-EL 620 (GAF, New York). The assay tubes consisted of PbTx-3 standard or ultracentrifuged lipoprotein fraction, anti-PbTx antiserum (1:4,000), and [^3^H]PbTx-3 (0.4 nM), in PBS (final assay volume of 500 μL). [^3^H]-PbTx-3 (21 Ci/mmol; 98.7% radiochemical purity by HPLC) was produced by sodium borohydride reduction of PbTx-2 by contract with Amersham Biosciences (Buckinghamshire, UK). An undetermined amount of [^3^H]-PbTx-9 double reaction product is likely present in the above preparation. The seven PbTx-3 standards ranged from 0.01 ng/mL to 1,000 ng/mL. We allowed the PbTx-3 standards and lipoprotein fractions to preincubate in buffer at room temperature with the anti-PbTx-3 antibody for 1 hr before the [3H]PbTx-3 tracer was added. The tubes were placed on a shaker (Titramax 100; Heidolph Instruments, Cinnaminson, NJ) and incubated 1 hr. We added Sac-Cel (Alpco Diagnostics, Windham, NH) to the assay tubes and filtered the bound antibody onto 25-mm glass fiber filters. Each assay tube was then rinsed with PBS (3 × 2 mL) using a 48-sample, Semi-Auto Harvester (Brandel, Gaithersburg, MD). We placed the filters in 5.0-mL Scintiverse (Fisher, Suwanee, GA) and counted the radioactivity on a Tri-Carb 3100TR Liquid Scintilation Counter (Packard-PerkinElmer, Wellesley, MA).

### Direct sandwich mouse albumin ELISA.

We used mouse albumin ELISA (enzyme-linked immunosorbent assay) quantitation kits (Bethyl Laboratories, Inc., Montgomery, TX) to quantify the albumin in each lipoprotein fraction. The plate was coated with a goat anti-mouse albumin antibody buffer. After a 60-min incubation, the plate was washed and then blocked with the postcoat solution. After incubation with the blocking postcoat solution for 30 min, we added the standard curve (7.8–10,000 ng/mL plus blank) and samples (lipoprotein fractions) to the plate and incubated it for 60 min. The samples were then washed off the plate and the horseradish peroxidase (HRP)-conjugated detection antibody (diluted 1:80,000) was added and incubated in the plate for 60 min. After washing the plate, we added TMB (3,5,3′,5′-tetramethylbenzidine), which reacts with the HRP to form a blue end product. We stopped the TMB reaction by adding 2 M H_2_SO_4_, which turned the blue product yellow. We then read the plate on a FluoStar plate reader (BMG Labtechnologies, Durham, NC) at 450 nm.

### [^3^H]PbTx-3–plasma albumin binding study.

Purified mouse albumin (100 mg) was solubilized in 1X PBS containing 137 mM NaCl, 8 mM Na_2_HPO_4_, 1.5 mM KH_2_PO_4_, and 2.7 mM KCl (all from Sigma Chemical Company). Each 300-μL 15-kDa molecular weight Spectra/Pro Float-A-Lyzer dialysis tube (Spectrum Laboratories Inc., Rancho Dominguez, CA) contained 282.5 μL of each albumin solution and 17.5 μL of a brevetoxin solution. The final concentration in each tube was 19, 38, and 76 mg/mL albumin in PBS, and 42.5 mg/mL albumin in mouse reference serum (Bethyl Laboratories, Inc.) with 29.8 ng/mL PbTx-3/[^3^H]PbTx-3 (32,000 cpm). The dialysis tubes were prepared and dialyzed in 1X PBS for a total of 22 hr with one buffer change. We counted the bound [^3^H]PbTx-3 in the dialysis tubes on a 1211 RackBeta Liquid Scintilation Counter (Wallac-PerkinElmer, Wellesley, MA).

### High-density lipoprotein SPA assay.

We modified high-density lipoprotein scintillation proximity assay (SPA) kits (Amersham Biosciences, Piscataway, NJ), using [^3^H]PbTx-3 as the radioligand, for direct brevetoxin binding to HDLs. Each experiment was composed of B_test_, NSB (nonspecific binding), and B_0_ groups. Every well contained 10 μL [^3^H]PbTx-3 (43 nM, 53,000 dpm) tracer and 10 μL SPA beads (poly-l-lysine coated YSi beads) with a final volume of 60 μL. Each B_test_ well contained 30 μL PbTx-3 (50 μM), 10 μL HDL (0.5 mg/mL) in addition to the tracer and beads. The NSB group contained 40 μL PBS and no HDL, whereas the B_0_ group contained 30 μL PBS and 10 μL HDL.

To determine percent inhibition, we used the following equation:


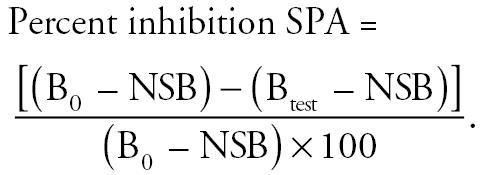


### Data analysis.

We determined all concentrations and EC_50_ (median effective concentration) values using Prism Graph Pad 4.0 (GraphPad Software, Inc., San Diego, CA).

## Results

We first determined whether mouse plasma has high molecular-binding components for brevetoxins. Mouse plasma and standard concentrations of albumin were spiked with [^3^H]PbTx-3, and unbound tracer was removed by 15-kDa molecular weight cutoff dialysis. Mouse albumin (20–80 mg/mL) retained a small fraction (< 6.8%) of the radiolabel after dialysis (Table 1). In contrast, mouse reference plasma containing 43 mg/mL albumin retained substantially more (39%) of the radiolabel after dialysis. These results indicated that the majority of brevetoxin binding in plasma is to component other than albumin.

**Figure 1:**
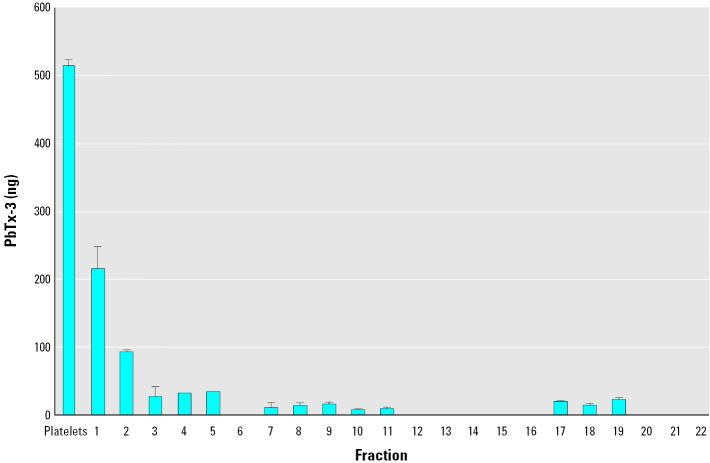
RIA determination of brevetoxin in lipoprotein fractions of spiked mouse plasma. Mouse plasma was spiked *in vitro* with PbTx-3 (100 ng/mL), and lipoprotein fractions were collected after iodixanol density gradient ultracentrifugation. RIA results are given as PbTx-3 equivalents (ng)/fraction and are mean ± SD from a single experiment.

We next examined if brevetoxin bound to lipoproteins in plasma. Mouse plasma was spiked with PbTx-3 and iodixanol gradient ultracentrifugation performed to separate lipoprotein particles based on density. The relative abundance of HDL and LDL/VLDL in the iodixanol gradient fractions was determined by agarose gel electrophoresis. We collected 26 0.5-mL fractions between the platelets and chylomicron bands. Fractions 1 and 2 had exclusively HDL, and fractions 3–20 showed decreasing HDL levels and increasing LDL/VLDL levels; fractions 21–26 had exclusively LDL/VLDL. Analysis of each fraction by radioimmunoassay indicated that the most dense lipoprotein fractions, those containing only HDLs, contained the greatest amount (85%) of brevetoxin immunoreactivity within the lipoprotein fractions (Figure 1).

**Figure 2:**
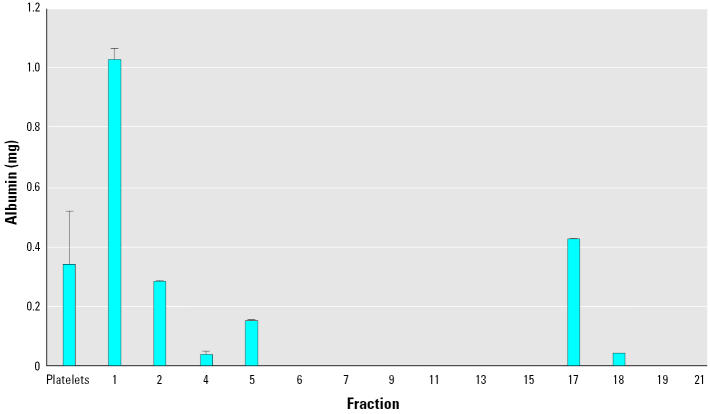
Determination of albumin in lipoprotein fractions of mouse plasma using direct sandwich ELISA. Results are shown as albumin (ng)/fraction and are mean ± SD from a single experiment.

We then examined the lipoprotein fractions to determine whether they may have been contaminated with concentrated levels of albumin. To determine if the albumin in these fractions contributed significantly to the binding of brevetoxin, each fraction was analyzed for the presence of mouse albumin using ELISA. Low but detectable albumin was present in some of the fractions, and distribution between fractions paralleled the distribution of brevetoxin (Figure 2). The total amount of albumin in the first two fractions (HDL) was 1.3 mg, a value much lower than the amount present in plasma (1.3 mg/4 mL vs. 40 mg/mL). Hence, although there was some contamination of HDL fractions with albumin, it was far less than could be accountable for brevetoxin binding.

**Figure 3:**
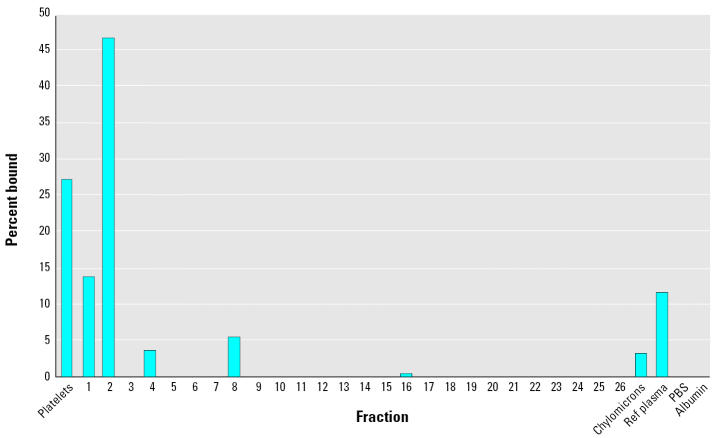
Determination of [^3^H]PbTx-3 association with plasma components with molecular weight > 100 kDa. ref, reference. Results are given as the percentage of PbTx-3 bound.

An independent approach was also taken to assure that the brevetoxin binding to the HDL fractions was not the result of other plasma carrier proteins. Each lipoprotein fraction, preincubated with [^3^H]PbTx-3, was dialyzed against a high molecular weight (100 kDa) cutoff membrane. The two homogenous HDL fractions once again showed the majority of binding of the lipoprotein fractions (Figure 3). This dialysis also permitted analysis of the platelet and chylomicron fractions for brevetoxin binding, and approximately 25% of the binding was to platelets and 2.5% was to the chylomicron fractions.

We confirmed our results using an HDL SPA. After incubating 6.5 pmol [^3^H]PbTx-3 with 5 μg purified human HDL under non-disturbed equilibrium conditions, 0.069 ± 0.015 fmol [^3^H]PbTx-3 bound per microgram of HDL. The binding of brevetoxin to HDL was reversible and saturable, as it was inhibited by 40% ± 7% in the presence of 50 μM unlabeled PbTx-3.

**Figure 4:**
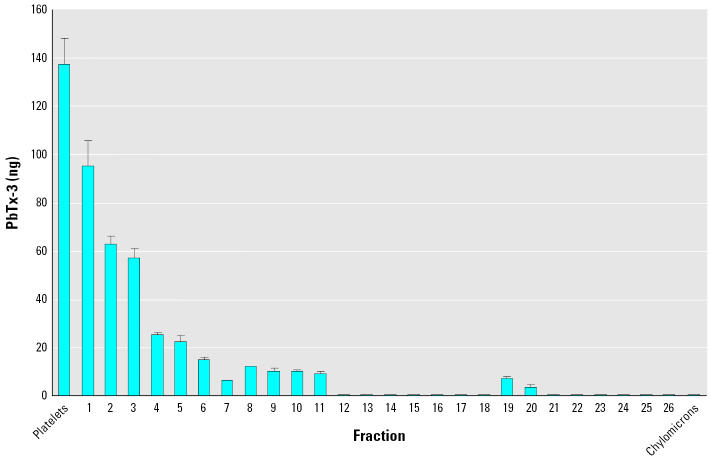
RIA determination of brevetoxin in lipoprotein fractions from mice exposed, *in vivo,* to PbTx-3. Mouse plasma was collected and pooled after being injected (ip) with PbTx-3 or vehicle. This plasma was then fractionated after iodixanol density gradient ultracentrifugation. The RIA results shown here are given as PbTx-3 equivalents (ng)/fraction and are mean values ± SD from a single experiment.

The final experiment was to expose mice *in vivo* to PbTx-3 to compare the distribution of brevetoxin in lipoproteins from our *in vitro* results to an *in vivo* exposure. Mice were treated ip with 310 μg/kg PbTx-3 for 30 min and their plasma was pooled. The plasma lipoproteins were separated and analyzed by RIA as described for the experiment presented in Figure 1. High levels of brevetoxin were found in the first two fractions containing exclusively HDLs, with decreasing levels of brevetoxin paralleling the decreasing levels of HDLs in fractions 3–11 (Figure 4). Substantial brevetoxin immunoreactivity was also associated with the platelet fraction.

## Discussion

### Binding to plasma/albumin.

Previous studies from our laboratory have found that brevetoxin achieves levels in whole blood more than a magnitude higher than its effective intrinsic concentration (i.e., the concentration required to bind voltage-gated sodium channels in nerve, muscle, or heart) (Woofter et al. 2003). Brevetoxin blood values remain between 25 and 30 nM for the first 12 hr in mice, whereas brevetoxin is effective at approximately 1–5 nM at site 5 of the voltage-gated sodium channel. This suggests that the majority of brevetoxin in blood may not be immediately biologically available and may be bound to cellular elements in blood or the fluid matrix (i.e., plasma). Because plasma contains specialized carrier proteins that may serve to transport brevetoxin to tissues, our investigation has focused on brevetoxin distribution in plasma. Dialysis of [^3^H]PbTx-3 spiked plasma revealed that 39% of the radiolabel was retained by plasma fractions of > 15 15kDa molecular weight. Of this, only < 6.8% of the binding was accounted for by binding to albumin (43 mg/mL) under these conditions. Albumin is a common binding protein for lipophilic compounds and has a well-characterized hydrophobic binding pocket. Whether albumin represents a greater percentage of binding under true equilibrium conditions in plasma cannot be resolved from these experiments; however, other factors in plasma provide nearly five times more binding capacity under the conditions used for this initial experiment.

### Binding to high-density lipoproteins.

Lipoproteins are predominantly recognized as plasma carrier particles for cholesterol and triglycerides. They are a heterogeneous class of protein–lipid aggregates traditionally classified by particle density and subsequently by apoprotein composition. We found that brevetoxin added to mouse plasma localized to HDL fractions after being purified by iodixanol gradient ultracentrifugation and characterized by agarose gel electrophoresis mobility. Because of some contamination of albumin from the adjacent platelet/fibrin fractions, we also confirmed using high molecular weight dialysis of [^3^H]PbTx-3 that the tracer was associated with plasma components > 100 kDa molecular weight. Additionally, we confirmed binding of [^3^H]PbTx-3 to human HDL under non-disturbed equilibrium SPA.

HDLs are the highest density class of lipoproteins. They are assembled in the interstitial space from aggregation of free phospholipids, cholesterol, and apo A1 proteins as discoidal “nascent” HDL particles. These particles then serve as a substrate for lecithin:cholesterol acyltransferase, leading to the esterification of cholesterol. The cholesterol ester localizes as an inner core and forces the HDL particles to form spheres, known as HDL_3_. The maturation of HDL_3_ to HDL_2_ results from the transfer of phospholipids, free cholesterol, and apoproteins, released from the lipolyzed VLDLs, to the HDL particle (Eisenberg 1984). These HDL_2_ particles are taken up by the liver and release cholesterol by a process known as reverse cholesterol transport. Hence, each class of HDL is composed of a phospholipid, cholesterol, and lipoprotein A1 outer membrane that successively accumulates triglycerides and cholesterol esters in an expanding inner core. The process by which HDLs accumulate cholesterol, first described by Bailey (1965), is the initial step (cholesterol efflux) of reverse cholesterol transport (Glomset 1973). This process is still not fully resolved; however, at least three separate mechanisms play a role in the uptake of cholesterol by HDL. The first mechanism is passive diffusion of cholesterol, which is a simple equilibrium of cholesterol between cellular and lipoprotein compartments. This process is not restricted to HDL, but is also operative for LDL and plasma-binding proteins such as albumin (Zhao and Marcel 1996), yet it is considered to be particularly well-suited to mature HDL_2_ because of the high cholesterol ester content. The other cholesterol-uptake mechanisms involve scavenger receptor B1 (SRB1) and the ATP-binding cassette subfamily, A member 1 (ABCA1) transporter.

### Binding to low-density lipoproteins.

Our data indicate that brevetoxin binds predominantly to the HDL class of lipoproteins in mouse plasma. However, this probably does not reflect the situation in other species, including humans, because mice, unlike normolipidemic humans, have small amounts of VLDLs and LDLs (as low as 20% vs. 70–80%, respectively) in the total lipoprotein pool (Grass et al. 1995). The low levels of VLDLs and LDLs present in mouse plasma may have reduced our ability to detect more substantial brevetoxin binding to these lipoprotein classes. Studies with several different classes of hydrophobic drugs, including cyclosporine A, amphotericin B, and nystatin, indicated that binding occurs to HDLs as well as to VLDLs and LDLs under *in vitro* conditions (Brajburg et al. 1984; Lemaire and Tillement, 1982; Wasan and Cassidy 1998). The mechanism for brevetoxin uptake under these conditions is also consistent with the passive diffusion of cholesterol. Certain drugs show a preference for HDL among the lipoproteins; however, this may be due to the high protein:lipid ratio of HDL (Cassidy et al. 1998; Kennedy and Wasan 1999; Wasan et al. 1997). Further studies will be needed with brevetoxins to determine the degree of selectivity for the toxin to the different lipoprotein classes, with particular reference to lipoprotein profiles representative of human plasma.

### Functional significance of brevetoxin binding to HDL.

PbTx-3 administered systemically to mice distributed to HDL fractions, indicating that brevetoxin binding to HDL may have physiologic significance. Under *in vivo* conditions, passive diffusion mechanisms likely play an important role in the uptake of brevetoxins to HDL. However, brevetoxins may also utilize two other mechanisms for cholesterol efflux. The first is SRB1-facilitated aqueous diffusion of cholesterol (Acton et al. 1996), a process that appears to involve the reorganization of membrane lipid domains that transfers cholesterol to various classes of lipoproteins (Rhainds and Brissette 2004). The second is the ABCA1 transporter, originally identified as a genetic defect in Tangier disease (Lawn et al. 1999). Regardless of the mechanism of brevetoxin uptake, hepatocytes would then promote transformation, conjugation, and reabsorption of brevetoxin into the bile. Excretion of a more polar metabolite would favor intestinal reabsorption and excretion via the kidneys into the urine. Recent studies have indicated that PbTx-2, the nonreduced precursor of PbTx-3, is particularly sensitive to transformation and conjugation in rats and is eliminated rapidly in the urine (Radwan et al. 2005) and hence would be a candidate to use this pathway.

### Basis for susceptibility and strategies for therapeutics.

The distribution of brevetoxins to lipoproteins may also indicate a basis for susceptibility in human populations, based on blood cholesterol-containing lipids. Indeed, studies with other compounds that bind to lipoproteins in plasma have shown that dyslipidemia alters drug distribution and enhances toxicity (Wasan and Cassidy 1998). In this regard, the distribution of brevetoxins by ip toxin exposure is not likely to reflect distribution of toxin by oral exposure, as was demonstrated for chlorpromazine and imipramine (Bickel 1975). After oral exposure, it is likely that absorption of brevetoxins across the intestinal mucosa will lead to their incorporation into chylomicrons. This may provide an important immediate distribution mechanism into the bloodstream and peripheral cells and then uptake by HDL or possibly LDL by one of the above-mentioned mechanisms of cholesterol efflux. Accordingly, lipoprotein profile or genetic variation in several of the mechanisms for reverse cholesterol transport (SRB1, ABCA1) may be expected to alter brevetoxin toxicokinetics and hence sensitivity to toxicity. Likewise, each of these pathways may provide a means for potential therapeutic intervention of brevetoxin in neurotoxic shellfish poisoning and the more prolonged effects of related polyether ciguatoxin for ciguatera fish poisoning. Indeed, the plasma levels of these toxins remain elevated (Bottein Dechraoui et al. 2005; Radwan et al. 2005; Woofter et al. 2003) and hence are continuously undergoing enterohepatic recirculation and intestinal reabsorption. In this light, another potential target for therapeutics is the enterohepatic recirculation pathway. Through the binding of bile salts and reduced intestinal reabsorption, bile acid sequestrants effectively disrupt this pathway and have been reported to reduce the symptoms of ochratoxin (Kerkadi et al. 1998, 1999). Accordingly, targeting both pathways for reverse cholesterol transfer and enterohepatic recirculation may ultimately lead to the development of therapies to mitigate the symptoms of neurotoxic shellfish poisoning and ciguatera fish poisoning.

## Figures and Tables

**Table 1:** Association of brevetoxin to mouse albumin and plasma.

Sample	Albumin (mg/mL)	PbTx-3 (% bound)
Mouse albumin	76	5.0
Mouse albumin	38	6.8
Mouse albumin	19	2.9
Reference plasma	43	38.7

Mouse plasma and different concentrations of mouse albumin were spiked with [^3^H]PbTx-3, and bound PbTx-3 was separated by 15 kDa molecular weight cutoff dialysis. Values are from individual sample dialysis bags that were analyzed in quintuplicate, with SEM < 5%.
